# A Wearable System Featuring Biomimetic Spatially Distributed Iontronic Sensing Array for Dynamic Monitoring of Deep Tissue Modulus

**DOI:** 10.1002/advs.202519009

**Published:** 2025-11-25

**Authors:** Zhenning Wang, Chaohua Fang, Ruoyu Sun, Chenghao Feng, Xiaoyuan Wang, Hao Bi, Yidan Wu, Jiangdong Gong, Yuyang Wang, Jiahao Guo, Yu Chang, Huizhi Wang, Tingrui Pan

**Affiliations:** ^1^ School of Biomedical Engineering, Division of Life Sciences and Medicine University of Science and Technology of China Hefei Anhui 230026 P. R. China; ^2^ Center for Intelligent Medical Equipment and Devices, Institute for Innovative Medical Devices, Suzhou Institute for Advanced Research University of Science and Technology of China Suzhou Jiangsu 215123 P. R. China; ^3^ Department of Sports Medicine, Shanghai General Hospital Shanghai Jiao Tong University School of Medicine Shanghai 200080 P. R. China; ^4^ National Center for Materials and Service Safety University of Science and Technology Beijing Beijing 100083 P. R. China; ^5^ Bionic Sensing and Intelligence Center (BSIC), Institute of Biomedical and Health Engineering, Shenzhen Institute of Advanced Technology Chinese Academy of Sciences Shenzhen Guangdong 518055 P. R. China; ^6^ Department of Precision Machinery and Precision Instrumentation University of Science and Technology of China Anhui Hefei 230026 P. R. China

**Keywords:** biomechanics, flexible electronics, health monitoring, muscle hardness, wearable sensor

## Abstract

Assessing soft tissue hardness is critical for disease diagnosis and motion monitoring. However, existing technologies are confined to skin‐level and quasi‐static measurements, leaving the dynamic behavior of deeper tissues, such as muscle, inaccessible. This study introduces a wearable system that enables dynamic monitoring of Young's modulus in multilayer tissues containing deep muscle (DMYD). Inspired by the spatial encoding strategy of human mechanoreceptors, DMYD integrates spatially distributed, high‐resolution, low‐detection‐limit iontronic sensing arrays with a load sensor to continuously capture the contact radius and contact force between a hemispherical indenter and the tissue, allowing real‐time and accurate modulus estimation based on Hertz contact theory. A simulation‐informed indentation strategy optimizes the accuracy of measurements in deep, multilayer tissues while minimizing wearing discomfort. In vitro experiments demonstrate that DMYD achieves high accuracy (>93%), supports dynamic operation, and remains robust to signal drift, sweat, and mechanical fatigue. In postoperative patients, its measurements correlate strongly with clinical edema indicators, while in healthy users, it tracks task‐dependent muscle hardness dynamics during rest, loaded elbow flexion, rope skipping, and stretching. Collectively, these results highlight DMYD as a promising platform for personalized and home‐based disease management, performance evaluation, injury‐risk warning, and training strategy optimization.

## Graphical abstract

A fully wearable system enables real‐time, dynamic, and accurate monitoring of Young's modulus in multilayer tissues containing deep muscle. It demonstrates high accuracy and robustness across simulations, benchtop validations, and human testing. It quantifies edema severity and tracks muscle stiffness during rest, loaded elbow flexion, rope skipping, and stretching, showing strong potential for personalized, home‐based health monitoring and performance optimization.

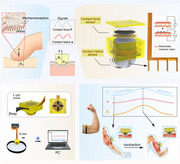

## 1 Introduction

Monitoring the hardness of multilayer soft tissues containing deep muscles provides physiologically and clinically meaningful information that cannot be captured from superficial tissues. Muscle hardness dynamically varies with neuromuscular activation, fatigue, and pathological conditions such as fibrosis, edema, muscular atrophy, and spasticity,^[^ ^1^, ^2^, ^3^, ^4^, ^5^ ^]^ whereas the hardness of superficial tissues such as skin and subcutaneous fat remains nearly unchanged during these processes. Abnormal elevation of deep tissue hardness can assist in the early detection and diagnosis of these disorders, while long‐term monitoring of hardness variations enables personalized adjustment of therapeutic strategies and improvement of intervention efficacy. During rehabilitation or exercise, monitoring regional changes in muscle hardness reflects training intensity and effectiveness, providing valuable feedback for optimizing training protocols. Overall, quantitative evaluation of deep tissue hardness is crucial for assessing musculoskeletal function, guiding personalized treatment, rehabilitation, and training decisions, and enabling long‐term postoperative monitoring. Young's modulus, as a geometry‐independent mechanical parameter^[^ ^6^ ^]^ is particularly suited for quantifying the hardness of irregularly shaped soft tissues. These insights underscore the necessity of developing wearable systems capable of dynamic and noninvasive modulus monitoring in deep tissues for applications in personalized and home‐based healthcare and performance optimization.

Existing methods for evaluating tissue modulus face notable limitations. Manual palpation is widely used in clinical practice but lacks quantitative rigor,^[^ ^7^ ^]^ and benchtop instruments like universal testing machines, though accurate,^[^ ^8^ ^]^ are impractical for in vivo use. Ultrasound elastography offers noninvasive options,^[^ ^9^ ^]^ but its reliability is often compromised by tissue heterogeneity, uncontrollable region‐of‐interest size, and operator dependence.^[^ ^10^ ^]^ Wearable platforms leveraging innovative sensing strategies have emerged to address these challenges. A vibration‐based system was proposed to estimate modulus from tissue deformation under vibrational forces,^[^ ^11^ ^]^ but slow signal stabilization and reliance on auxiliary tools for deep tissue measurement limit its real‐time and in‐depth applicability. Indentation‐based techniques, grounded in contact mechanics such as the Hertz model,^[^ ^12^ ^]^ provide a direct and geometry‐independent approach by measuring compressive force and contact radius or depth. While effective at micro‐ and nanoscale (e.g., atomic force microscopy and nanoindentation),^[^ ^13^ ^]^ these techniques typically rely on benchtop setups. Recent advances in flexible electronics have enabled wearable force sensors, but estimating contact depth or radius remains difficult. Approaches using strain‐gauge^[^ ^12^ ^]^ or triboelectric sensors^[^ ^14^ ^]^ often require manual operation and only provide discrete measurements, with structural constraints limiting their use to superficial tissues. To date, accurately quantifying the modulus of composite deep tissues in vivo remains challenging, while the demand for real‐time dynamic monitoring imposes additional technical hurdles. Their integration into a fully wearable platform remains unachieved.

**Figure 1:**
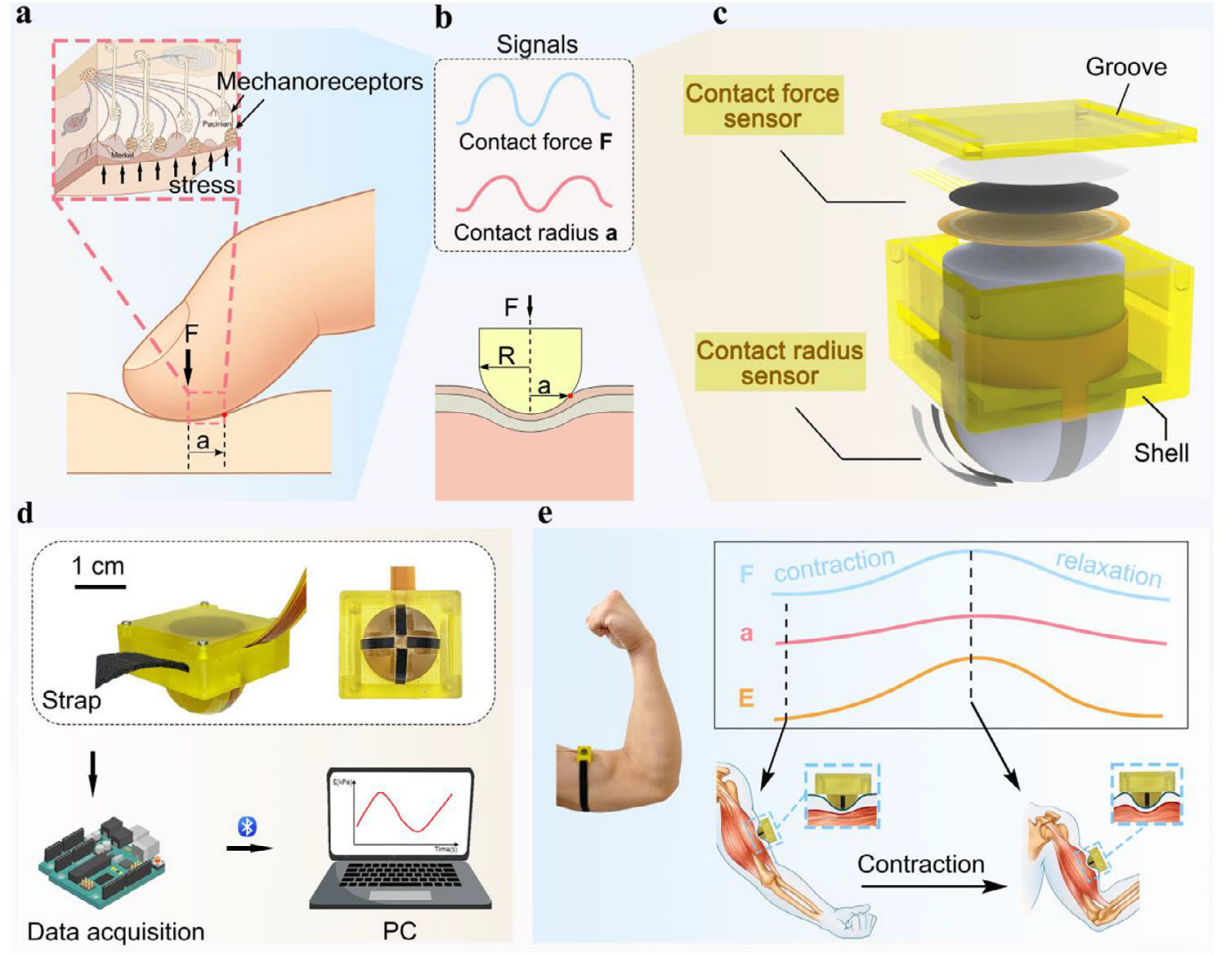
Overview of the DMYD system. a) Schematic illustrating how human fingers perceive material hardness through tapping. b) Illustration of the hemispherical indenter pressing into a representative composite soft tissue and the key signals captured. c) Diagram of the wearable sensing module. d) Schematic of the integrated system architecture. e) Illustration of how DMYD continuously captures key signals during a representative dynamic activity.

Human finger pads are densely packed with mechanoreceptors, such as Merkel discs and Pacinian corpuscles (Figure 1a), enabling sensitive tactile perception. These receptors are distributed across distinct locations and depths, and are innervated by fast‐conducting nerve fibers. A single nerve fiber can innervate multiple receptors, allowing spatially distributed signals to be integrated in real time. This coordinated organization gives rise to receptive fields with fine spatial resolution, allowing the finger pad to detect pressure magnitude and localize contact area in real time. By integrating pressure, spatial, and temporal cues, the finger pad perceives tissue hardness and its dynamic variations during palpation, with touch strategies adapted to probe different layers, from light contact for superficial tissues to deeper indentation for underlying structures.^[^ ^15^ ^]^

Inspired by the natural sensing strategy of the finger pad, we developed a wearable system for real‐time dynamic monitoring of Young's modulus in multilayer soft tissues containing deep muscle (DMYD). Mimicking the finger pad's sensing mechanism, DMYD synchronizes the measurement of contact force and contact radius at the interface between a hemispherical indenter and the target tissue. The indenter integrates four uniformly distributed iontronic pressure‐sensing arrays with high spatial resolution (0.4 mm) and low detection limit (< 0.2 kPa), which continuously reconstruct the contact radius by registering the number of activated sensing units. This design mimics the neural strategy in which a single nerve fiber encodes signals from spatially distributed mechanoreceptors. Simultaneously, a highly reliable pressure sensor positioned above the indenter measures contact force, enabling modulus estimation through the Hertz contact model. In addition, inspired by the finger pad's adaptive indentation strategy, we performed finite element simulations to explore how indentation depth, indenter size, and tissue hardness affect estimation accuracy and user comfort when probing multilayer tissues. In vitro tests demonstrated high accuracy in quantifying Young's modulus of synthetic homogeneous and multilayer heterogeneous PDMS phantoms (> 93%) and porcine muscle specimens (93.6%). In postoperative limbs, DMYD measurements correlated strongly with thigh circumference, the clinical standard for edema evaluation, supporting its potential for home‐based monitoring. In human trials, DMYD tracked deep tissue mechanics in both static and dynamic states, distinguishing sex‐related differences and capturing real‐time modulus variations across activities including elbow flexion, rope skipping, and stretching. It revealed task‐specific temporal profiles, detected hardness increases after rope skipping, and reductions following stretching. Collectively, these findings establish DMYD as a robust platform for quantitative, real‐time, and dynamic assessment of deep tissue mechanics across pathological and physiological conditions, with broad potential in disease management, performance evaluation, and training strategy optimization.

## 2 Results and Discussion

### 2.1 Working Principle and System Design

Guided by the finger pad's spatial‐encoding rationale introduced in the Introduction (Figure 1a),^[^ ^15^ ^]^ we designed a bimodal intelligent wearable sensing system (DMYD) featuring a hemispherical indenter (Figure 1c). During compressive interaction with the target tissue, the system simultaneously captures two types of signals: contact force and contact radius (Figure 1b). Both signals are governed by the indenter diameter and the tissue's Young's modulus. This relationship can be well characterized by the Hertz contact model^[^ ^12^ ^]^ in homogeneous tissues, allowing the estimation of tissue modulus using Equation (1).

(1) $$E=\frac{3}{4}\left(1-\nu^{2}\right)FRa^{-3}$$

where *E* is the Young's modulus of the tested material, *ν* is the Poisson's ratio (assumed to be 0.5 for soft biological tissues^[^ ^16^ ^]^), *F* is the contact force, *R* is the diameter of the indenter, and *a* is the contact radius.

Based on this principle, two sensing modules were integrated into the system: a contact force sensor and a contact radius sensor. Operating independently yet synchronously, these sensors continuously capture key interaction signals. The contact force sensor is a highly repeatable and accurate piezoresistive element covering the top of the indenter, designed to capture the full contact pressure. The contact radius sensor, representing the core innovation of the system, consists of four flexible iontronic pressure sensor arrays that are evenly distributed along the hemispherical surface and aligned axially (Figure 1c,d). It emulates the finger pad's spatial distribution of mechanoreceptors and the way a single afferent pools signals from multiple receptors, enabling real‐time decoding of pressure‐active locations. The symmetrical layout of these arrays minimizes calculation errors arising from potential tilt between the device and tissue surface during dynamic motions, thereby enhancing robustness and measurement accuracy.

Inspired by the hierarchical sensing organization of the human finger pad, the DMYD system translates key biological sensing principles into engineering implementations. Specifically, the spatially distributed iontronic pressure‐sensing arrays mimic the distribution of mechanoreceptors (e.g., Merkel discs and Pacinian corpuscles) responsible for local pressure detection and fine spatial encoding. The shared signal‐integration circuit mirrors the neural convergence process, in which a single afferent neuron integrates information from multiple receptors to construct a unified tactile response. Furthermore, the simultaneous acquisition of contact force and contact radius parallels the integration of pressure and spatial cues in natural tactile perception, enabling the system to infer tissue hardness in real time. The indentation strategy, examined through finite element simulations (see Section 2.3 for details), reproduces the finger pad's ability to determine indentation depth according to tissue depth, thereby ensuring accurate probing of deep soft tissues. Collectively, this bioinspired design allows DMYD to achieve both high sensitivity and reliable mechanical quantification, bridging the gap between human tactile perception and artificial modulus sensing.

Two sensing modalities were employed in this system, namely resistive sensing for contact force and iontronic sensing for contact radius, because they serve different functional roles. The contact force signal quantifies the magnitude of contact strength, requiring high accuracy, repeatability, and reliability. In contrast, the contact radius signal characterizes the spatial extent of contact, demanding high sensitivity and a low detection limit to capture subtle pressure variations near the contact edges. The resistive sensing mechanism was chosen for force detection owing to its structural simplicity, mature fabrication process, and excellent reproducibility. Commercially available materials can readily satisfy the quantitative measurement requirements of this component. Conversely, the iontronic sensing mechanism, which leverages the ultrahigh specific capacitance arising from the electrical double layer effect, enables substantial signal variation under minute deformations.^[^ ^17^ ^]^ This property offers superior sensitivity and an ultralow detection limit, making it particularly suitable for tracking the evolution of the contact radius.

To support sensor integration and wearability, a cylindrical extension above the indenter anchors the sensor wires, while the structural shell includes grooves on the top and sides for wire routing and strap fixation. Before testing, the device can be secured to relaxed limbs using a strap, ensuring full contact between the indenter and the tissue surface (Figure 1e). During activities such as elbow flexion, changes in muscle hardness and thickness lead to fluctuations in contact force and radius, which are continuously tracked by the sensor arrays to yield a real‐time modulus readout (Figure 1e).

The system integrates the sensing modules with a data acquisition card and a host computer to form a complete wearable platform (Figure 1d). Sensor signals are continuously collected by the data acquisition card and wirelessly transmitted to the host computer via Bluetooth. The host computer processes these signals in real time to compute the Young's modulus, enabling continuous readout of tissue mechanics during dynamic functional tasks.

### 2.2 Sensor Fabrication and Performance Characterization

#### 2.2.1 Contact Force Sensor

**Figure 2:**
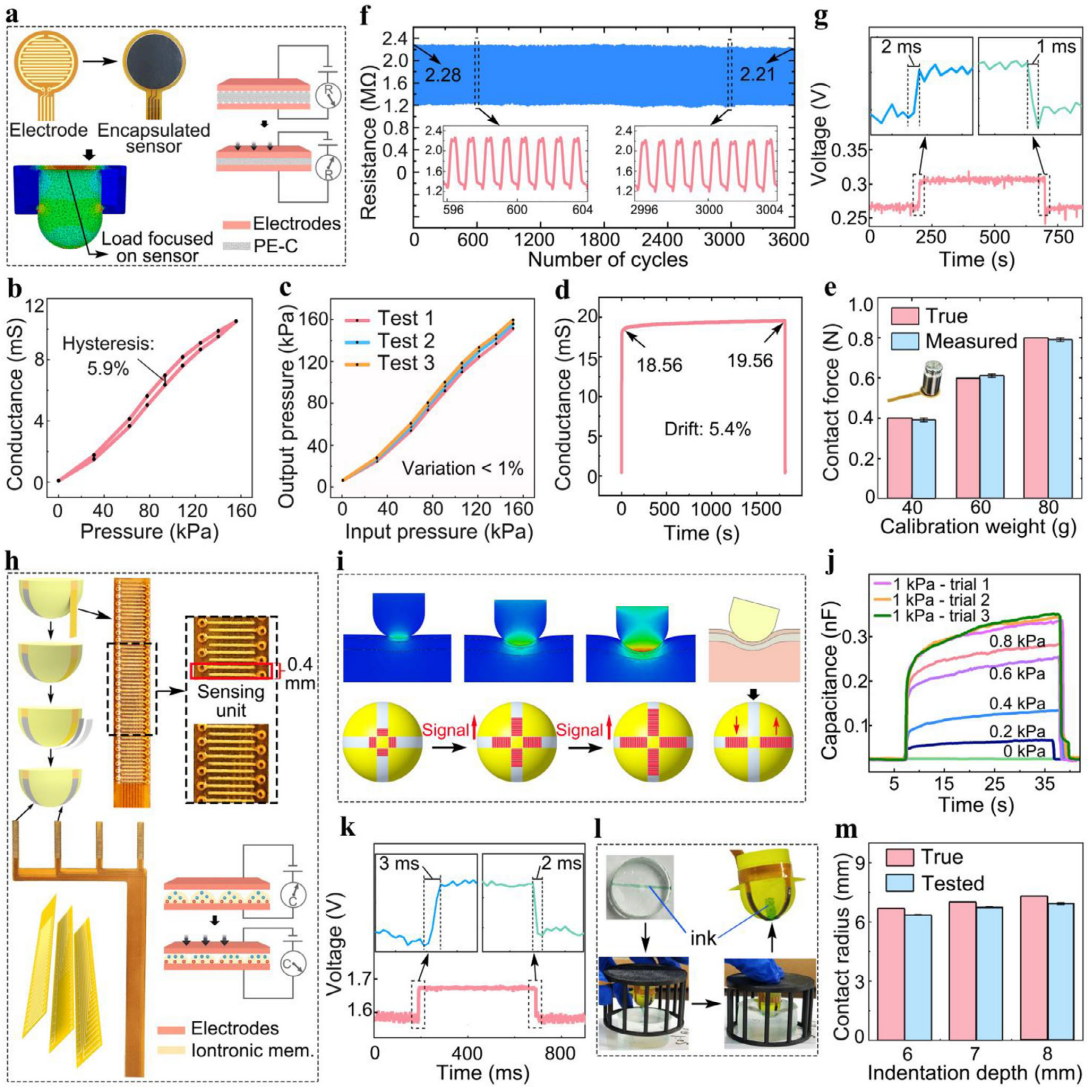
Sensor fabrication and characterization. a) Schematic of the electrode layout and the encapsulated contact force sensor with its sensing mechanism, together with a finite element simulation illustrating the force transmission path. b) Loading‐unloading pressure‐response curves of the contact force sensor, showing minimal hysteresis. c) Measured pressure versus applied (true) pressure across three repeated trials. d) Drift performance of the contact force sensor. e) Measurement accuracy of the contact force sensor. f) Output resistance of the contact force sensor over 3600 loading cycles. g) Dynamic response of the contact force sensor. h) Schematic of the structural design, fabrication process, and sensing mechanism of the contact radius sensor. i) Illustration of signal variations of the contact radius sensor under different loading conditions. j) Capacitance response of the contact radius sensor to stepwise pressure inputs of different magnitudes, demonstrating its reliable low detection limit. k) Dynamic response of the contact radius sensor. l) Experimental setup for evaluating the measurement accuracy of the contact radius sensor. m) Measurement accuracy of the contact radius sensor.

The contact force sensor consists of three layers: an electrode layer, a functional layer, and an encapsulation layer (Figure 1c). The electrode layer is composed of copper‐plated polyimide (PI) film and features an interdigitated (fork‐shaped) architecture (Figure 2a), while the functional layer is composed of conductive polyethylene (PE) film (LINQSTAT, CAPLINQ, The Netherlands). A Polyurethane (PU) film serves as the encapsulation layer. Upon the application of pressure, deformation of the functional layer decreases its resistance, allowing the electrical response (voltage) to be transduced into a load signal. The contact force sensor is designed to fully cover the top surface of the indenter. Finite element analysis confirmed that this configuration effectively captures the total force generated at the sensor‐tissue contact interface (Figure 2a), enabling accurate force measurement during operation.

Experimental evaluations demonstrated that the contact force sensor possesses high linearity (nonlinearity = 3.4%), low hysteresis (5.9%; Figure 2b), and excellent repeatability (coefficient of variation = 0.9%; Figure 2c), indicating its capability for accurately capturing signals with varying magnitudes. The drawback of high hysteresis is common among flexible pressure sensors due to the inherent viscoelasticity of soft materials. In previously reported studies, hysteresis values of typical flexible sensors generally range from 10% to 20%,^[^ ^18^ ^]^ while the best‐performing ones can reach ≈3%.^[^ ^19^, ^20^ ^]^ The hysteresis of 5.9% observed in our device is therefore not the lowest reported but still represents a comparatively low and competitive level of performance. Drift testing revealed only a 5.4% variation in output conductance over 30 min of continuous loading (Figure 2d), underscoring the sensor's high operational stability. In addition, static load tests using calibration weights of 0.4, 0.6, and 0.8 N confirmed a measurement error of less than 1% (Figure 2e), validating the sensor's accuracy and reliability in force measurement across a range of applied loads.

Furthermore, the sensor demonstrated excellent mechanical fatigue resistance, maintaining a stable resistance output over 3600 loading‐unloading cycles at 1 Hz under a 6 N load (Figure 2f), highlighting its robustness for long‐term dynamic monitoring in wearable applications.

To assess its dynamic response characteristics, the sensor was subjected to rapid loading using a piezoelectric beam operating at 1000 Hz, and exhibited a response time within 2 ms (Figure 2g), which is sufficient for real‐time monitoring of daily activities such as walking, stair climbing, and running. In this test, voltage variations induced by changes in resistance or conductance were recorded using a data acquisition card (NI USB‐6361, National Instruments, USA) with a sampling rate of 1000 Hz, instead of directly reporting resistance values, since conventional LCR meters, although capable of measuring resistance and capacitance, cannot achieve such a high sampling rate.

#### 2.2.2 Contact Radius Sensor

The contact radius sensor also adopts a sandwich structure (Figure 1c), comprising a copper electrode, an iontronic functional layer, and a PU encapsulation. The iontronic layer confers supercapacitive behavior, and its microstructure amplifies contact area changes under compression, producing marked capacitance variations that directly reflect the applied pressure (Figure 2h). Inspired by the spatial distribution of mechanoreceptors within the finger pad and their array‐like pattern of neural innervation, the contact radius sensor is designed to integrate four iontronic sensing arrays with high spatial resolution and a low detection limit, enabling sensitive and accurate detection of the contact radius. The spacing between adjacent sensing units was set to 0.4 mm (Figure 2h), similar to the subdomain‐level spatial resolution of mechanoreceptors in the finger pad.^[^ ^15^ ^]^ These sensing units are conformally distributed along the hemispherical surface of the indenter, allowing the maximum detectable contact radius to match the radius of the indenter. As the indenter presses deeper into the tissue, the number of sensing units registering non‐zero pressure increases (Figure 2i). These units are referred to as “activated units”, analogous to mechanoreceptors in the finger pad that are stimulated by pressure. By continuously counting the number of activated units, the corresponding contact geometry can be inferred in real‐time. To account for potential tilt between the device and the tissue surface during movement, which may lead to asymmetric activation of sensing units on opposite sides, we averaged the number of activated units across all four arrays to minimize detection errors (Figure 2i).

To achieve high spatial resolution in the arrangement of sensing units while ensuring conformal integration with the curved surface of the indenter, the physical width of each array was minimized to enhance flexibility and reduce bending‐induced noise. This design imposed significant challenges for signal routing. To address this, we implemented a 1D array based on a three‐layer stacked electrode architecture (Figure 2h), where the electrodes on either side of each sensing unit were routed using an alternating (odd‐even) and pairwise grouping strategy, effectively constraining the overall sensor width to within 2 mm. This enabled tight and stable attachment to the curved hemispherical indenter surface.

To achieve a low detection limit, we employed a highly sensitive iontronic functional layer consisting of indium tin oxide (ITO)‐based iontronic membrane, allowing the sensor to capture subtle pressures near the edge of the indenter‐tissue interface. Finite element simulation showed that the minimum pressure at the contact edge was ≈3 kPa. Experimental results confirmed that the sensor could detect pressures as low as 0.2 kPa (Figure 2j), which is well below the simulated threshold. Repeated tests (n = 3) showed that the relative error of the instantaneous capacitance jump upon applying a 1 kPa load was less than 3%, further confirming the reliability of detection in the sub‐kilopascal regime. Under no applied pressure, the sensor's output capacitance drifted by less than 0.01 nF within 30 min, which is far smaller than the instantaneous capacitance change induced by a 0.2 kPa load. This result indicates that the sensor remains stable over long‐term use and would not be falsely triggered when no pressure is applied. In addition, the sensor exhibited fast response and recovery times within 3 ms (Figure 2k), enabling real‐time tracking of transient changes in contact radius during dynamic activities.

To evaluate the accuracy of contact radius detection, a green ink trace was applied across the diameter of a cylindrical PDMS elastomer, and the indenter was pressed into it to a fixed depth using a custom mold with a preset height (Figure 2l). The actual contact radius (derived from the inked area on the indenter) was compared with the value measured by the DMYD system. The sensor achieved a high detection accuracy of 95.3% (Figure 2m), confirming its reliability in capturing the true contact radius.

### 2.3 Finite Element Analysis of Sensor‐Tissue Interaction for Accurate Measurement and Comfortable Wearability

**Figure 3:**
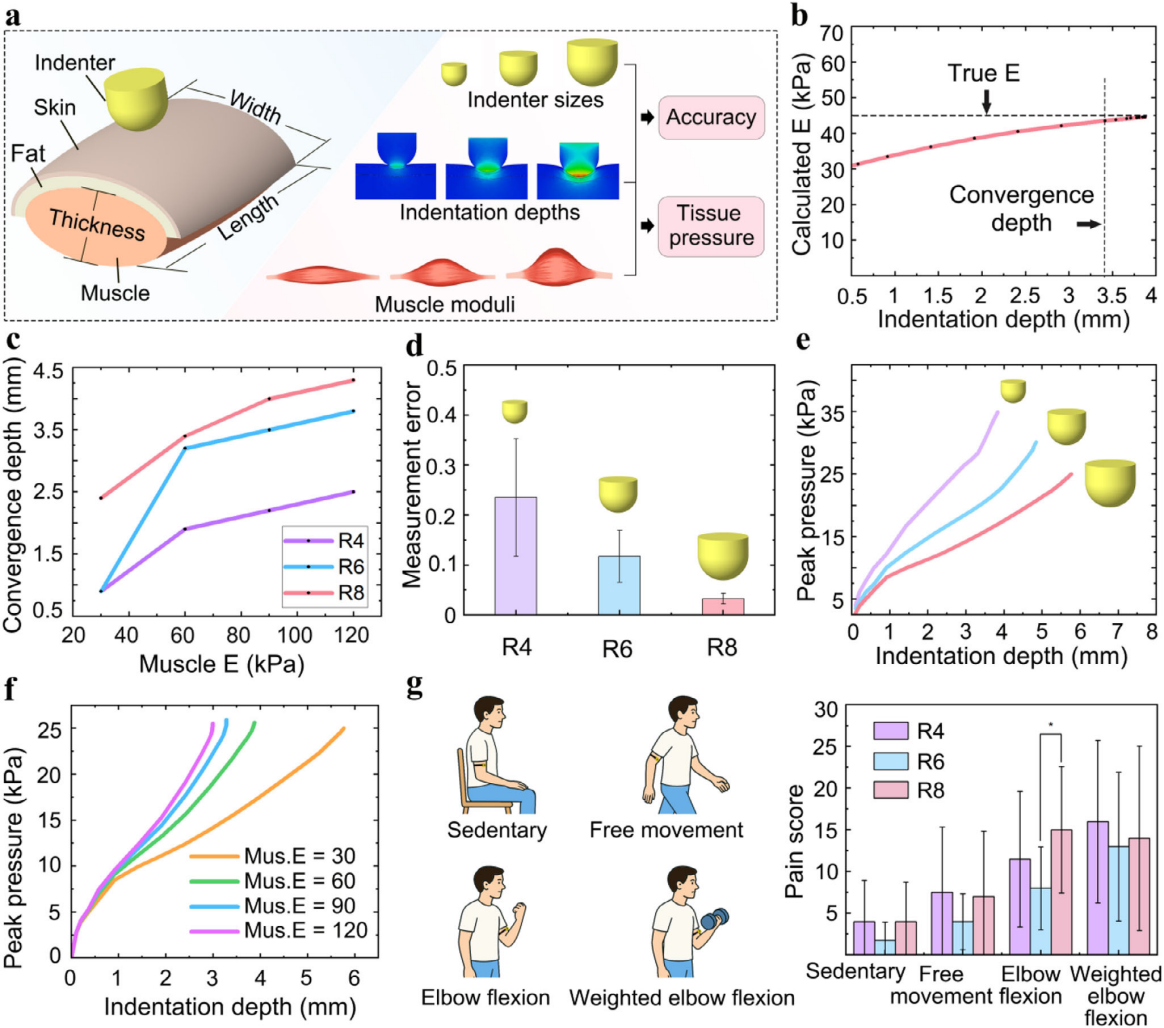
Finite element analysis of indenter‐tissue interactions for accurate measurement and comfortable wearability. a) Schematic of the finite element model with varying indenter diameters, indentation depths, and muscle moduli. b) Calculated Young's modulus (E) as a function of indentation depth. c) Effect of indenter size and muscle modulus on indentation convergence depth. d) Effect of indenter size on measurement error. e) Combined Effects of indenter size and indentation depth on peak tissue pressure. f) Combined effect of muscle modulus and indentation depth on peak tissue pressure. g) In vivo evaluation of subjective pain scores under various physical activities with DMYD using different indenter sizes.

While the Hertz model assumes that the measured material is homogeneous, most tissues of interest in pathological and movement monitoring are inherently multilayered and heterogeneous, typically comprising skin, subcutaneous fat, muscle, and bone. As noted earlier, when probing deeper tissues, the finger pad usually applies a greater indentation to capture more realistic mechanical properties. Based on this, we hypothesized that shallow indentation depths reflect only the hardness of superficial tissues, whereas deeper indentations are required to capture the mechanical contributions of underlying layers. To test this hypothesis, we developed a finite element model of a skin‐fat‐muscle composite. Given the substantially higher hardness of bone, it was excluded from the model, and the bottom of the muscle layer was fully fixed to mimic the near‐rigid constraint of the underlying bone. The model systematically evaluated three core variables: indentation depth (0–8 mm), indenter diameter (4, 6, and 8 mm), and muscle Young's modulus (30, 60, 90, and 120 kPa) corresponding to the physiological hardness range during relaxation, walking, running, and stair ascent.^[^ ^21^, ^22^ ^]^ For each simulation, two primary evaluation metrics, measurement accuracy and wear comfort (peak tissue pressure), were quantified to assess the combined effects of the core variables (Figure 3a).

We found that as indentation depth increased, the calculated Young's modulus gradually converged to the true value (Figure 3b), indicating that deeper indentation enables the measurement to better reflect the effective modulus of the composite tissue. We defined the convergence depth as the depth at which a further 0.5 mm indentation results in less than 0.2% variation in the computed modulus.

Convergence depth increased with both indenter diameter and muscle modulus (Figure 3c). We speculate that a smaller indenter diameter may lead to faster convergence of the contact radius, resulting in earlier convergence of the measured modulus. Additionally, when deeper tissues are stiffer, a greater indentation depth may be required to sufficiently involve them in the mechanical response. Measurement accuracy also improved with a larger indenter diameter (Figure 3d). This is likely because larger indenters generate lower local contact strain, better satisfying the small‐strain assumption of the Hertz model^[^ ^23^ ^]^ while their greater indentation depth enables better integration of modulus contributions from deeper layers. Among all conditions tested, the R8 indenter exhibited the largest convergence depth (4.3 mm) and also achieved the highest measurement accuracy (93%). Considering that indentation depth may fluctuate with muscle contractions during dynamic movement, and that deeper indentation improves accuracy, we determined that an indenter with a diameter of 8 mm should have a height of at least 6–8 mm to ensure adequate initial indentation and stable performance during use. The displayed measurement errors may arise from limitations inherent to finite element modeling. Alternatively, they may reflect the fact that multilayered biological tissues violate the Hertz model's homogeneity assumption and that the indentation range exceeds the model's small‐strain limits. Nevertheless, despite these limitations, the Hertz model remains the most widely adopted framework for characterizing the mechanical properties of elastomers in localized testing.^[^ ^23^ ^]^

We also found that peak tissue pressure increased with greater indentation depth and smaller indenter diameter (Figure 3e), probably due to larger compressive force and more concentrated stress, respectively. Higher muscle modulus also produced higher peak pressure (Figure 3f). This may be attributed to the fact that achieving the same indentation depth in stiffer tissues likely requires greater force. Notably, all peak pressures remained below the pain threshold of human skin (≈400 kPa^[^ ^24^ ^]^), with a maximum of 35 kPa, suggesting low risk of discomfort within the simulated indentation range.

Although finite element analysis helps to uncover the fundamental mechanisms of device‐tissue interaction, severe mesh distortion typically causes computational failure at indentation depths beyond 5.5 mm. To further verify user comfort in real applications, an in vivo study involving 12 healthy participants (6 males, 6 females, aged 23–32) was conducted using three indenter configurations: R4 (4 mm height), R6 (6 mm height), and R8 (8 mm height). Participants were instructed to wear the device for 1 min under each of the following conditions: sedentary, free movement, elbow flexion, and weighted elbow flexion at 36% of maximal voluntary contraction, representing the maximum muscle activation during daily activities (Figure 3g).^[^ ^22^ ^]^ No significant differences in VAS pain scores (range 0–100)^[^ ^25^ ^]^ were observed across most conditions, except between sedentary and weighted elbow flexion (6 ±  2 vs 14  ± 1, p < 0.05), and no significant differences among indenters were observed, except between R6 and R8 during elbow flexion (Figure 3g). However, in all cases, pain scores remained below 15 (mean 9), indicating minimal discomfort during use.

Based on these findings, the R8 indenter was selected as the optimal configuration due to its superior accuracy and comfort for evaluating deeper composite tissues. The final indenter height was set to 8 mm to ensure sufficient initial indentation during use.

### 2.4 Validation of System Accuracy on PDMS Samples and Porcine Muscle Specimens

**Figure 4:**
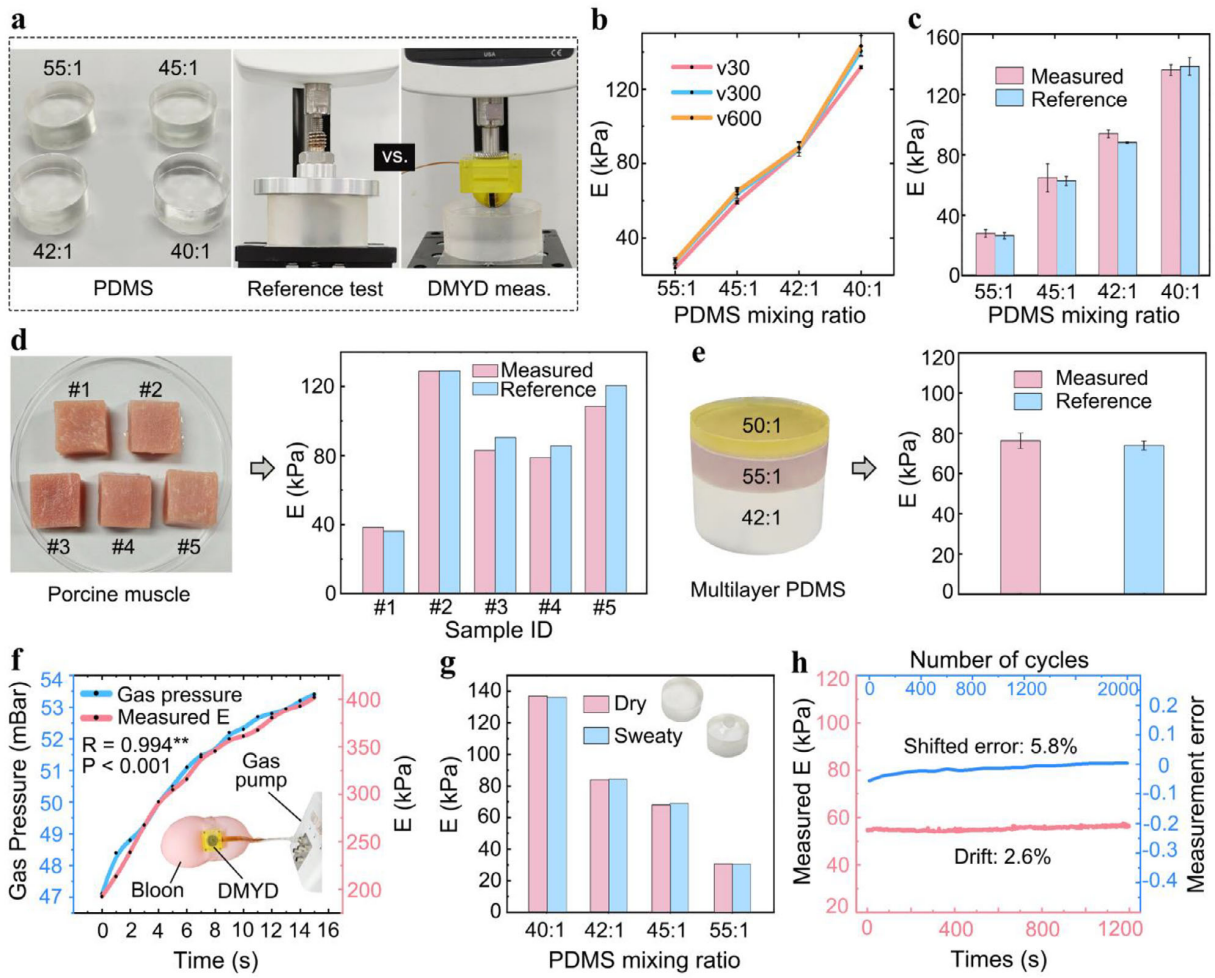
Evaluation of measurement accuracy and system performance. a) Photograph of homogeneous PDMS samples with varying moduli and schematic of modulus measurement using the reference system and DMYD. Black labels in the left panel indicate the base‐to‐curing agent ratio for each sample. b) Reference Young's modulus (E) of PDMS samples at different loading rates. v30: 30 mm min^−1^. c) Comparison between DMYD‐measured and reference moduli across homogeneous PDMS samples. d) Photographs of porcine muscle specimens and comparison of Young's moduli measured by DMYD versus the reference system. e) Photograph of a multilayer heterogeneous PDMS phantom and corresponding modulus comparison between the measurements by DMYD and the reference system. f) Changes in DMYD‐measured modulus with increasing air pressure during continuous gas inflation of a balloon, demonstrating the system's dynamic measurement capability. g) Performance comparison of DMYD on dry and sweaty PDMS samples. h) Time‐dependent variation in measured modulus showing the system's drift characteristics, and variation in measurement error with loading cycles indicating the system's fatigue resistance.

To validate the accuracy of the DMYD system, homogeneous PDMS elastomers with varying base‐to‐curing agent ratios were prepared to mimic soft tissues of varying hardness, corresponding to Young's moduli of ≈30, 60, 90, and 120 kPa (Figure 4a). Reference Young's moduli were obtained using a universal testing machine (Series F505IM, MARK‐10, USA), the widely recognized gold standard for modulus measurement, at loading rates of 30, 300, and 600 mm min^−1^ (Figure 4a). No significant differences were observed across loading rates (Figure 4b), indicating the low viscoelasticity of the PDMS samples. The same samples were then tested with the DMYD system using 8 mm indentations under identical loading rates. Paired *t*‐test analysis showed no significant differences between DMYD‐measured and reference moduli (p = 0.31), with a mean measurement accuracy of 93.5% across all samples (Figure 4c).

Further validation was performed on five porcine muscle specimens at a loading rate of 300 mm min^−1^. Each specimen was cut into a standardized block (≈30 mm × 30 mm × 22 mm) (Figure 4d) for gold‐standard testing. Again, no significant difference was observed between DMYD and reference measurements (p = 0.13), with a mean accuracy of 93.6% (Figure 4d).

DMYD was further evaluated on a multilayer heterogeneous PDMS phantom with distinct layer moduli, achieving an accuracy of 96.8% (76 ± 3 kPa measured by DMYD vs. 74 ± 1 kPa obtained by universal testing; Figure 4e).

Collectively, these results demonstrate the robust capability of DMYD to accurately estimate Young's modulus in both synthetic phantoms and biological soft tissues, as well as in homogeneous and multi‐layer heterogeneous materials. Compared with previous studies, such as that by Cui et al.,^[^ ^12^ ^]^ which reported accuracies above 80% for soft materials below 1 MPa, the improved performance of DMYD may be attributed to the use of independent, high‐precision sensors for simultaneous contact force and contact radius measurements.

### 2.5 System Performance

To evaluate the dynamic sensing performance of the system, a gas pump was used to continuously inflate a balloon, thereby generating a steadily increasing internal pressure, while the DMYD system simultaneously monitored the changes in the balloon's Young's modulus. The results demonstrated that the measured Young's modulus values increased in synchrony with the applied pressure, with a correlation coefficient exceeding 0.99 (p < 0.01; Figure 4f), indicating the superior capability of the DMYD system for dynamic monitoring.

To assess the potential impact of sweat induced by physical activity on system accuracy, artificial sweat (pH 5.5; Shenzhen Zhongwei Equipment Co., Ltd., China) was applied to the surfaces of PDMS samples. The Young's modulus measured under sweaty conditions showed no significant difference from that under dry conditions (p > 0.05; Figure 4g), confirming that the system remains accurate even in the presence of surface moisture.

To evaluate the long‐term stability of the DMYD system, it was first worn onto the biceps brachii of a subject in a relaxed and motionless state. After 20 min of continuous wear, the measured Young's modulus increased by only 2.6% compared to the initial value (Figure 4h), indicating minimal signal drift over time. Second, the DMYD system was mounted on a universal testing machine and subjected to repeated indentation‐retraction cycles on a PDMS sample with an indentation depth of 8 mm. The Young's modulus was recorded every 100 cycles and compared with the reference value. After 2000 cycles, the measurement error shifted by 5.8% (Figure 4h), which demonstrates excellent fatigue resistance and mechanical stability. Lastly, the system was used to measure the modulus of PDMS samples at different hardness levels over a continuous 15 day period, showing stable readings with a total variation of less than 7% across the entire test duration (Figure S3, Suporting Information).

The DMYD system was systematically benchmarked against existing methods across key metrics (**Table** **1**). Compared with existing systems, DMYD demonstrates competitive accuracy while uniquely enabling in vivo modulus detection of multilayer composite tissues containing muscle, primarily owing to our in‐depth investigation of device‐tissue interaction mechanisms. Furthermore, DMYD markedly accelerates acquisition speed (20 ms vs. 3 s in Cui et al.^[^ ^12^ ^]^ and 1 min in Song et al.^[^ ^11^ ^]^), thereby allowing real‐time tracking of rapid hardness variations during dynamic activities. Its fully wearable design further enables continuous monitoring under daily‐life conditions. Notably, compared with previous Hertz‐model‐based studies on Young's modulus detection,^[^ ^12^, ^14^ ^]^ our work builds upon accurate contact pressure measurement and further introduces a high‐density, spatially distributed iontronic pressure sensor array to enable precise and real‐time measurement of the contact radius. Moreover, unlike several prior studies that directly correlate sensor signals with hardness via machine learning,^[^ ^26^, ^27^ ^]^ our analysis is grounded in well‐established theoretical derivation and physical computation. Future integration of data‐driven approaches may further enhance the system's performance. Collectively, these advances establish DMYD as a robust platform for in vivo, high‐precision, real‐time, dynamic, and practical assessment of deep composite soft tissue mechanics.

**Table 1:** Comparison of the DMYD system with existing methods for in vivo quantification of soft tissue Young's modulus based on key performance metrics.

Methods	Accuracy	In vivo applicability to muscle‐containing multilayer tissues	Dynamic monitoring	Real‐time monitoring	Acquisition time	Wearable
Universal testing	100% (Gold standard)	×	×	×	Several minutes	×
Ultrasound elastography	< 50%^[^ ^28^ ^]^	√	√	√	Tens to hundreds of ms	√
Strain sensing under vibrational loading^[^ ^11^ ^]^	> 95%	× (Depths >2 cm may require ultrasound and different sensor sizes, limiting accuracy and wearable applicability)	√	×	1 min	×
Self‐locking structure for controlled indentation depth and strain sheet for load measurement^[^ ^12^ ^]^	> 80%	×	×	×	3 s	√
Triboelectric sensor for indentation depth measurement^[^ ^14^ ^]^	96%	×	×	×	> 15 s	×
DMYD	93%	√	√	√	20 ms	√

### 2.6 Quantification of Joint Edema Following Ligament Reconstruction Surgery

Ligament injuries are among the most common sports‐related conditions, and reconstruction is the standard treatment.^[^ ^29^ ^]^ However, postoperative joint edema frequently occurs, leading to pain, stiffness, and reduced mobility that hinder rehabilitation.^[^ ^30^ ^]^ Quantitative assessment of edema can support personalized interventions such as cryotherapy, kinesio taping, or medication^[^ ^31^ ^]^ and also enables home‐based monitoring to reduce demand on medical resources. The standard approach for assessing joint edema involves measuring limb circumference with a tape.^[^ ^32^ ^]^ However, surgical wounds and dressings, which require daily changes, can impair measurement accuracy. Additionally, patients must lift the affected limb during assessment, increasing procedural difficulty and reducing patient compliance.

**Figure 5:**
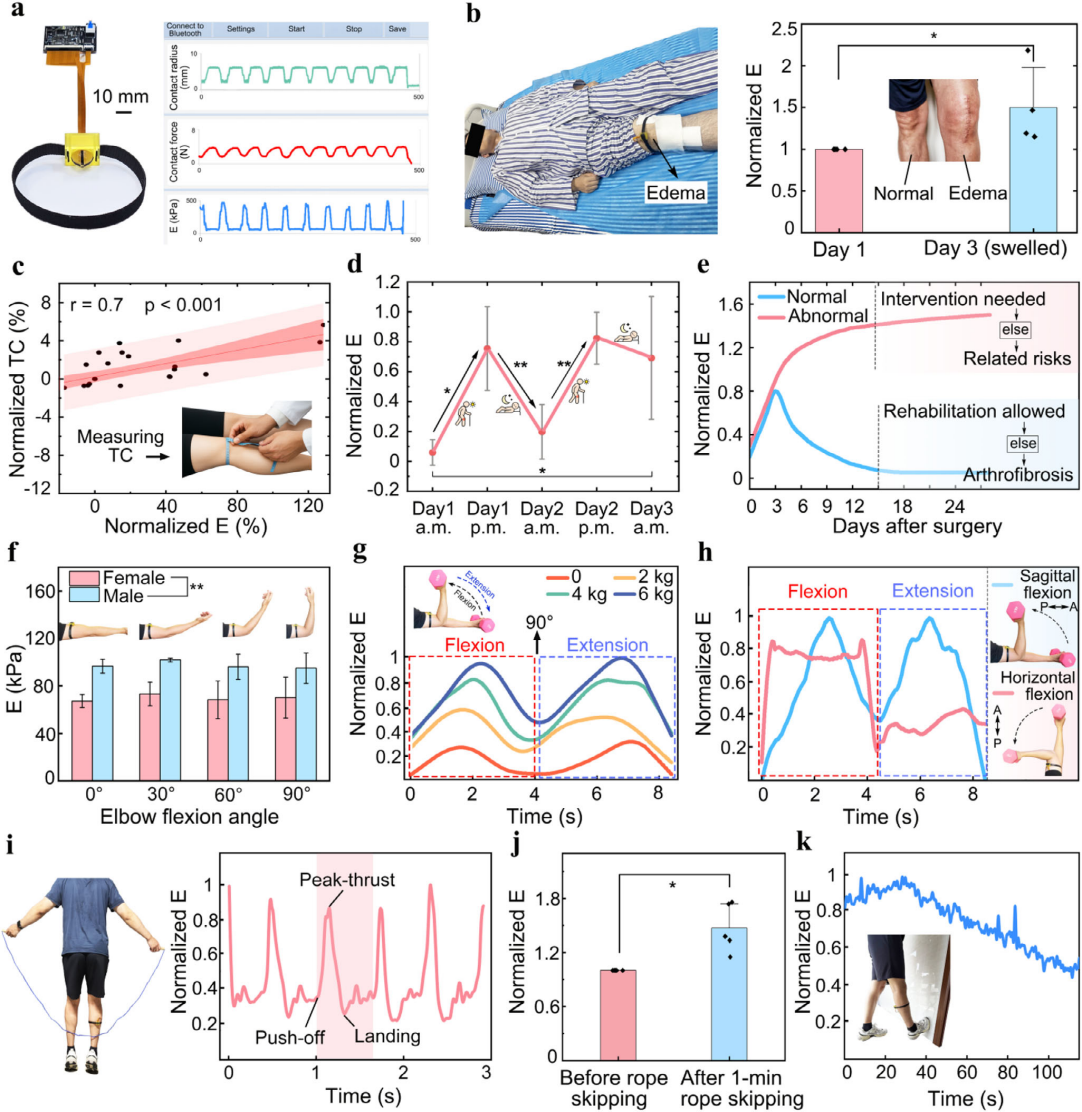
Representative applications of the DMYD system. a) Photograph showing the DMYD system in use during real‐world scenarios. b) Photograph of DMYD being used to assess joint edema after ligament reconstruction surgery, accompanied by a bar chart comparing normalized periarticular tissue Young's modulus (E) between postoperative day 1 and day 3, with statistical analysis. ^*^: Significant difference with p < 0.05. c) Correlation between normalized E measured by DMYD and thigh circumference (TC). Red line: fitted regression; dark pink: prediction interval; light pink: confidence interval. d) Postoperative changes in normalized E over time, showing regular increases in the afternoon and decreases in the morning. ^**^: Significant difference with p < 0.01. e) Illustrative curves showing potential DMYD applications in monitoring normal versus abnormal postoperative edema trends and guiding related clinical decisions. f) Comparison of tissue E at the biceps site between male and female participants across different elbow flexion angles. g) Variation of normalized tissue E at the biceps site during elbow flexion and extension under different loads. h) DMYD‐measured normalized biceps tissue modulus changes during loaded elbow flexion‐extension in the sagittal and horizontal planes, highlighting its potential for evaluating exercise effects and optimizing training strategies. i) Real‐time changes in the normalized tissue E at the gastrocnemius site during rope skipping. Pink band: one complete cycle. j) Comparison of the normalized tissue E at the gastrocnemius site before and after rope skipping. k) Real‐time changes in the normalized tissue E during static stretching of the gastrocnemius.

We explored the feasibility of using the DMYD system for quantitative monitoring of joint edema following ligament reconstruction. Figure 5a presents a photograph of the DMYD system in a real‐world application, consisting of a wearable sensing module, a data acquisition card, and a host computer. Five patients undergoing anterior cruciate ligament (ACL) reconstruction were recruited (34 ± 10 years old; 3 males and 2 females). On postoperative days 1, 2, and 3, limb circumference (measured with a tape) and periarticular soft tissue modulus (measured using the DMYD system) were assessed at 10:00 a.m. and 6:00 p.m. at a standardized location 1 cm above the superior border of the patella, with an indentation depth of 8 mm and the knee in full extension (Figure 5b). This indentation depth ensures that the composite soft tissue containing muscle, with a total thickness exceeding 28 mm, can be accurately measured, as validated by the finite element analysis. To better illustrate intra‐individual variations, all measured values were normalized to those obtained at 10:00 a.m. on day 1. Statistical analysis revealed a significant increase in normalized tissue modulus on postoperative day 3 compared with day 1 (p < 0.05; Figure 5b), suggesting the presence of knee swelling during this period, which is consistent with previous reports.^[^ ^33^ ^]^ A strong correlation was found between the normalized thigh circumference and tissue modulus (Pearson's r = 0.7, p < 0.001; Figure 5c), suggesting that DMYD measured periarticular soft tissue modulus can serve as an effective indicator of joint edema severity. Furthermore, the patients exhibited consistent temporal changes in the normalized modulus, showing lower values in the morning and higher values in the evening (Figure 5d). This trend may be attributed to daytime activities, such as sitting to eat or walking to the bathroom, which increase joint loading and exacerbate edema, while nighttime rest likely reduces inflammation and promotes fluid resorption.

In future work, the DMYD system could support the development of quantitative, edema‐based criteria to determine the optimal timing for postoperative rehabilitation or secondary intervention (Figure 5e). For example, if the patient's periarticular tissue modulus follows a normal trajectory, peaking at three days after surgery and decreasing significantly by two weeks,^[^ ^34^ ^]^ rehabilitation training could be initiated once a specific threshold is reached, thereby avoiding joint arthrofibrosis from delayed mobilization.^[^ ^35^ ^]^ In contrast, if the tissue modulus continues to rise abnormally and exceeds a defined threshold at a certain time point, urgent pharmacological or surgical intervention may be warranted to avoid delayed rehabilitation, excessive opiate use, blood transfusion, and related risks.^[^ ^36^ ^]^ These DMYD‐based strategies require further validation in large‐scale clinical studies to establish standardized decision‐making criteria. Such applications would enable personalized edema management, reduce postoperative complications, clarify the link between rehabilitation timing and joint functional recovery, and ultimately inform the development of evidence‐based rehabilitation protocols.

### 2.7 Dynamic Monitoring of Human Muscle Contraction, Stiffening, and Relaxation

The system was also applied to in vivo tests for monitoring muscle dynamics during different activities. During these tests, the device was worn on the arms and legs with an initial indentation depth of 8 mm. It should be noted that, during dynamic activities, the indentation depth fluctuated with muscle hardness changes. The recorded indentation depths during rest, elbow flexion, rope skipping, and static stretching ranged from 4.7 to 7.8 mm.

#### 2.7.1 Monitoring Biceps Brachii Activity During Rest and Elbow Flexion‐Extension Exercises

The biceps brachii plays a critical role in upper‐limb motor control, particularly in elbow flexion and forearm supination. Accurate monitoring of its activity is essential for assessing upper‐limb function and evaluating rehabilitation and exercise outcomes.^[^ ^37^ ^]^ Traditional methods, such as surface electromyography, provide valuable insights into neural activation patterns but fall short in capturing mechanical changes within the muscle itself. Real‐time tracking of biceps mechanical behavior can therefore serve as a valuable complement, offering a more comprehensive understanding of muscle performance.

To evaluate muscle hardness at rest, six healthy participants (Age: 25 ± 1 years old; height: 165 ± 9 cm; weight: 61 ± 17 kg, 3 males and 3 females) were recruited. The tissue modulus at the biceps brachii site was measured while the participants remained relaxed at elbow flexion angles of 0°, 30°, 60°, and 90°. The measured values ranged from 51.5 to 103.7 kPa, with males exhibiting significantly higher values than females (97.4 ± 7.7 vs 69.7 ± 10.9 kPa; p < 0.001; Figure 5f), which may partly account for their generally greater muscle strength. However, no significant differences were observed across elbow flexion angles, likely because the muscles remained unactivated in all tested positions, resulting in similar mechanical properties of the multilayer composite tissue.

We then assessed tissue modulus during continuous elbow flexion and extension in the sagittal plane under progressively increasing loads (0, 2, 4, and 6 kg). All measured values were normalized to the maximum value obtained throughout the activity to ensure consistency with the presentation format used in the clinical tests. The normalized modulus peaked at an elbow flexion angle of 45° and reached minima at 0° and 90° (Figure 5g). These low‐modulus positions reflect postures where the load is partially supported by the table or bone structure, reducing the demand for active muscular engagement. As the load increased, tissue modulus increased accordingly, but the rate of increase gradually plateaued. This trend may reflect a physiological ceiling in muscle hardness during maximal voluntary contraction, suggesting that the biceps were approaching their maximal mechanical output under high‐load conditions.

In another test, we compared the changes in the normalized biceps tissue modulus during loaded elbow flexion and extension exercises performed in the sagittal and horizontal planes (Figure 5h). Although the load was identical in both exercises, the patterns of hardness increase caused by muscle contraction differed markedly. In the sagittal‐plane exercise, tissue hardness showed a distinct increase followed by a decrease during both flexion and extension. By contrast, during the horizontal‐plane task, hardness remained high and nearly unchanged throughout elbow flexion, but stayed low throughout extension. This difference may be explained by the primary function of the biceps brachii in generating torque to flex the elbow, whereas elbow extension is mainly driven by the triceps brachii. However, during elbow extension in the sagittal plane, the biceps must keep contracting to counteract the reverse torque generated by the dumbbell's weight, thereby maintaining a constant extension speed. In contrast, in the horizontal plane, this torque is primarily resisted by muscles in the lateral upper arm, allowing the biceps to remain fully relaxed during extension. These results suggest that strength training targeting the biceps should be performed in the sagittal plane, as horizontal‐plane flexion exercises are considerably less effective. Furthermore, the findings highlight that different training movements produce distinct muscle activation patterns, which can be effectively captured by DMYD. Abnormal activation patterns may indicate incorrect exercise execution. Therefore, DMYD holds strong potential for assessing exercise effectiveness and optimizing training strategies.

#### 2.7.2 Monitoring Gastrocnemius Activity During Rope Skipping and Static Stretching

The gastrocnemius muscle plays a critical role in lower‐limb mobility during activities involving push‐off, such as walking, stair ascending, and jumping.^[^ ^38^ ^]^ Monitoring its mechanical response during such activities is crucial for understanding muscle dynamics and supporting clinical evaluation, rehabilitation planning, and performance assessment in both medical and athletic contexts.

We found that when the DMYD was worn over the gastrocnemius region of a subject's lower limb during rope skipping, the tissue modulus exhibited clear cyclic patterns (Figure 5i). Each cycle began with a sharp increase in modulus as both feet pushed off to lift the body, followed by a rapid decrease during the airborne phase, reaching a minimum just before landing. Fluctuations were observed after landing and before the next push‐off, reflecting ongoing muscle adjustments to maintain postural stability. Statistical analysis of five healthy subjects (Age: 25 ± 1 years old; height: 175 ± 6 cm; weight: 76 ± 14 kg, 5 males) showed a significant increase in tissue modulus normalized to the initial value after 1 min of rope skipping (p < 0.05; Figure 5j), likely due to muscle edema following explosive exertion.^[^ ^39^ ^]^ After rope skipping, passive calf stretching was performed with the forefoot placed against a wall, while DMYD continuously monitored tissue modulus. As shown in Figure 5k, the modulus began to decrease after 30 s of sustained stretching, and the rate of decline slowed after ≈100 s. These results suggest that DMYD can capture rapid, cycle‐specific changes in muscle mechanics during rapid dynamic activities such as rope skipping, as well as gradual modulus alterations during prolonged static stretching. This capability enables detailed characterization of muscle function under both high‐intensity and recovery conditions, offering potential applications in individualized sports performance assessment, exercise optimization, injury prevention, and rehabilitation monitoring.

## 3 Limitations

This study has several limitations. First, although we have preliminarily demonstrated the feasibility of using the DMYD system for quantitative monitoring of postoperative tissue edema, further research is required to enable formal clinical translation. Such translation typically necessitates large‐scale clinical studies to establish quantitative thresholds that define the “safe zone” and determine the optimal timing for postoperative rehabilitation or secondary interventions. Moreover, studies with larger cohorts are warranted to compare the therapeutic efficacy of different treatments, such as pharmacological therapy, cryotherapy, and kinesio taping. Second, the in vivo experiments were conducted only on young, healthy participants. The measured values may differ when older populations or individuals with obesity are included. Third, the modulus estimation in this study relies on the classical Hertz contact model, which assumes homogeneous, isotropic, and small‐strain conditions. These assumptions are partially violated in multilayer biological tissues, potentially introducing systematic bias in hardness estimation. To address this limitation, future studies may employ advanced data‐driven correction strategies, such as machine learning or physics‐informed neural networks, to model the nonlinear mapping between contact parameters and tissue hardness under heterogeneous and large‐deformation conditions. Alternatively, tissue‐specific finite element models that mimic the macroscopic geometry and microscopic structure of target tissues could be developed to investigate their mechanical responses under realistic loading. Insights from such simulations may inform the construction of corrected or hybrid Hertz‐type models, thereby enhancing the physical fidelity and predictive accuracy of modulus estimation in complex biological tissues. Fourth, the proposed technology is tailored to muscle‐containing multilayer composite tissues and related applications, but is not intended for scenarios requiring quantification of the hardness of a specific homogeneous tissue layer. Furthermore, the current DMYD prototype was fabricated using 3D printing and small‐batch manual assembly, serving primarily as a proof‐of‐concept demonstration. Although the system shows excellent accuracy and robustness in laboratory and in vivo tests, standardized large‐scale manufacturing has not yet been implemented. Future work will focus on developing scalable fabrication and integration processes to enhance reproducibility, reduce cost, and facilitate broader clinical and commercial deployment.

## 4 Conclusion

In this study, we present a wearable system that integrates four spatially distributed iontronic sensing arrays with a load sensor to enable real‐time, dynamic monitoring of Young's modulus of multilayer composite tissues containing deep muscle (DMYD). Finite element simulations, in vitro validation, and in vivo tests demonstrate high accuracy, low wearing discomfort, and superior robustness under static and dynamic conditions. In addition, DMYD correlates with the clinical edema proxy of thigh circumference and resolves task‐specific temporal hardness profiles during daily activities and resistance training. These capabilities position DMYD as a promising platform for home‐based clinical monitoring, performance evaluation, risk prediction, and individualized training optimization.

## 5 Experimental Section

### Sensor Design and Fabrication—Finite Element Analysis of Sensor‐Tissue Interaction

3D models of the skin‐fat‐muscle composite were created in SolidWorks (Dassault Systèmes, France) and meshed using HyperMesh (Altair Engineering, Japan). Finite element analyses were conducted in Abaqus/CAE (Simulia, Inc., United States). The dimensions and material properties of the skin,^[^ ^40^, ^41^, ^42^, ^43^ ^]^ fat,^[^ ^40^, ^44^, ^45^, ^46^, ^47^, ^48^, ^49^ ^]^ and muscle layers^[^ ^21^, ^22^, ^44^, ^45^, ^50^ ^]^ were defined based on literature data for the upper arm at the biceps region, as summarized in Table 2. The indenter was modeled as a biocompatible resin with a Young's modulus of 2 GPa. Tie constraints were applied at the skin‐fat and fat‐muscle interfaces to simulate anatomical integrity, while the bottom surface of the muscle was fixed to simulate the near‐rigid behavior of the underlying bone. Indentation was simulated by pressing the spherical indenter into the tissue model with displacement control, and the resulting contact force F was recorded at various indentation depths *h*. The effective Young's modulus was estimated based on a modified Hertz model (Equation (2)^[^ ^12^ ^]^), and the true modulus was derived using Equation (3).

(2) $$E=\frac{3}{4}\left(1-\nu^{2}\right)FR^{-\frac{1}{2}}h^{-\frac{3}{2}}$$

(3) $$\frac{1}{E^{*}}=\sum_{i=1}^{n}\frac{V_{i}}{E_{i}\times V_{total}}$$

where E^*^ denotes the effective Young's modulus of the composite tissue, *V_i_* represents the thickness of the i‐th tissue layer, *E_i_* is the Young's modulus of the i‐th tissue layer, and *V_total_* is the total thickness of the composite tissue.

**Table 2:** The dimensions and material properties of skin, fat, and muscle are defined in the finite element model.

	Skin	Subcutaneous fat	Muscle	Indenter
Length (mm)	50.0	50.0	50.0	–
Width (mm)	59.8	57.3	50.5	–
Thickness (mm)	1.25	3.4	24.1	–
Young's modulus (kPa)	24	18	30 ‐ 120	2 × 10^6^
Poisson's ratio	0.5	0.5	0.5	0.35
Element size (mm)	1.5	1.5	1.5	0.25

A mesh convergence test was conducted to ensure a balance between computational accuracy and efficiency. An indenter with an 8 mm radius and a muscle modulus of 60 kPa was used in this test. The element size was progressively refined until further reductions produced negligible changes in the calculated effective Young's modulus. The finalized mesh parameters used in the following simulations are listed in **Table** **2**.

In the formal simulations, the indenter was pressed into the composite tissue model under displacement control, and the resulting contact force was continuously recorded to calculate the effective Young's modulus using Equation (2).

### Sensor Design and Fabrication—Fabrication of the Ionic Membrane

A 30 wt.% thermoplastic polyurethane (TPU) solution was prepared by dissolving TPU 75A and TPU 5778 in a mixed solvent of isophorone and dimethyl sulfoxide (DMSO) (3:1 by mass). The solution was stirred in a sealed beaker at 70 °C and 210 rpm until fully dissolved and visually homogeneous. The resulting TPU solution was then mixed with 1‐ethyl‐3‐methylimidazolium bis (trifluoromethylsulfonyl) imide ([EMIM][TFSI]) ionic liquid and indium tin oxide (ITO) powder in a predetermined mass ratio. After manual stirring for initial homogenization, the mixture was processed in a defoaming machine with two cycles of 2 min stirring and 2.5 min vacuum degassing, yielding a uniform ITO composite ionic gel. A 50 µm‐thick transparent PET substrate was fixed on a screen‐printing platform. The ionic gel was cast onto a 250‐mesh screen and patterned via screen printing using a doctor blade. The printed membrane was dried at 110 °C for 20 min and cooled to room temperature, yielding the final ITO composite ionic membrane.

### Sensor Design and Fabrication—Signal Processing and Calibration

The raw resistance signal from the contact force sensor was calibrated to contact force (F, in N) using an experimentally derived calibration curve. For the contact radius, the iontronic sensing arrays did not directly convert the electrical signals into a radius value. Instead, the number of sensing units showing a significant increase in capacitance was first identified. The contact radius (a, in mm) was then calculated based on the known spatial configuration and geometry of the sensing arrays, as detailed in Note S1 (Suporting Information). Subsequently, Young's modulus (E, in kPa) was calculated from the measured contact force (F) and contact radius (a) using the Hertz contact model (Equation (1)). For consistency across figures, force was reported in N, contact radius in mm, and modulus in kPa.

### In Vitro Validation—Fabrication of PDMS Samples

PDMS samples were prepared in cylindrical form by mixing 40 g of PDMS base with 1 g of curing agent (base‐to‐curing agent ratio: 40:1). The mixture was stirred for 3 min using a glass rod to ensure uniformity, then degassed in a centrifugal defoaming machine for 5 min. A custom cylindrical mold (inner diameter: 40 mm, height: 20 mm) was evenly coated with mold release agent, and the degassed mixture was slowly poured into the mold to avoid bubble formation. The mold was cured in an oven at 60 °C for 24 h, after which the PDMS cylinder was carefully demolded. Additional samples were fabricated using base‐to‐curing agent ratios of 42:1, 45:1, and 55:1, yielding four PDMS elastomers of identical dimensions (40 mm diameter, 20 mm height) for subsequent experiments.

### In Vitro Validation—Universal Testing to Obtain Reference Young's Moduli

Specimens were placed on the stage of a universal testing machine (Series F505IM, MARK‐10, USA; Figure 4a). The upper loading platen was lowered until it just contacted the specimen, after which axial compression was applied at preset crosshead speeds (30, 300, and 600 mm min^−1^) to a maximum displacement of 40% of the specimen's initial height. The resulting compressive force‐displacement curve was recorded, and the reference Young's modulus was calculated from the linear region of the curve using Equation (4).

(4) $$E=\frac{\Delta F*L_{0}}{\Delta L*S_{0}}$$

where *E* denotes the specimen's Young's modulus, Δ*F* denotes the change in compressive force within the linear region of the force‐displacement curve, Δ*L* denotes the corresponding displacement change within the linear region, *L*_0_ denotes the specimen's initial height, and *S*_0_ denotes its initial cross‐sectional area.

### Statistical Analysis

Pre‐experimental measurements from three samples were used for a power analysis to estimate the required sample size using PASS 15 (PASS, Rijswijk, Netherlands), with α of 0.05 and power of 90%. Data normality was assessed using the Kolmogorov‐Smirnov test. Paired‐sample t‐tests were conducted using IBM SPSS Statistics 27 (SPSS Inc., Chicago, USA) to evaluate statistical differences among comparison groups. A significance level of P < 0.05 was set.

### Ethical Approval

All experiments involving human participants were approved by the Institutional Review Board (IRB) of the University (Approval Code: BE‐2024‐PJ‐001). Written informed consent was obtained from all subjects prior to participation.

## Conflict of Interest

The authors declare no conflict of interest.

## Supporting information



Supporting Information

## Data Availability

All data are available in the main text.
