# Supplementary material for: A Wearable System Featuring Biomimetic Spatially Distributed Iontronic Sensing Array for Dynamic Monitoring of Deep Tissue Modulus
Source: Adv Sci (Weinh). 2025 Nov 25;13(8):e19009. doi: 10.1002/advs.202519009 (PMC12884733; doi:10.1002/advs.202519009)
Supplement: Supplementary file 1 — Supporting Information [file ADVS-13-e19009-s001.docx]

**Supplementary Information for**

**A Wearable System Featuring Biomimetic Spatially Distributed Iontronic Sensing Array for Dynamic Monitoring of Deep Tissue Modulus**

**Table of Contents**

Figure S1. Time-drift curve of the contact radius sensor...............................................2

Note S1. Calculation of contact radius from the activation pattern of the iontronic sensing array...................................................................................................................3

Figure S2. Schematic illustration of the principle for contact radius calculation..........3

Figure S3. Variation in the measured modulus of PDMS over 15 consecutive days................................................................................................................................4


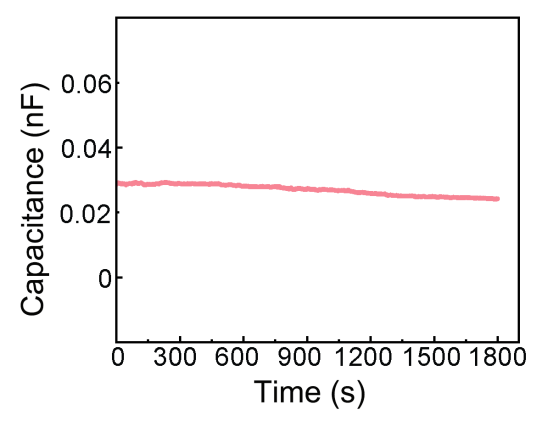


**Figure S1.** Time-drift curve of the contact radius sensor.

**Note S1. Calculation of contact radius from the activation pattern of the iontronic sensing array**

The contact radius sensing module consists of four iontronic pressure-sensing arrays uniformly distributed along the hemispherical surface of the indenter, with each array containing 28 sensing units (Figure S2). The center-to-center spacing between adjacent sensing units is 0.4 mm. When a sensing unit is subjected to pressure, its capacitance exhibits a step-like increase. The data acquisition system counts, at each time point, the number (N) of activated sensing units that show a sudden capacitance change, and the corresponding contact radius (a) is then calculated according to the following equations:

$\theta=\pi*\frac{L+N*0.4}{\pi*R}$ （S1）

$\sin\theta=\frac{a}{R}$ （S2）

Here, R denotes the radius of the indenter, and L represents the arc length from the array edge to the central axis of the indenter. By combining Equations (S1) and (S2), Equation (S3) can be derived as follows:

$a=R*\sin\frac{L+N*0.4}{R}$ （S3）


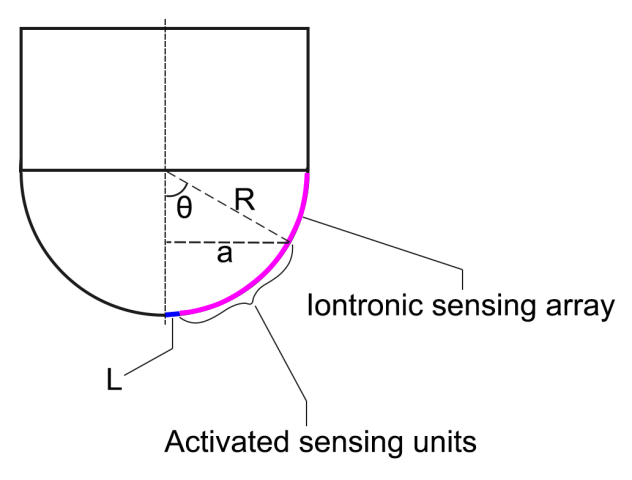


**Figure S2.** Schematic illustration of the principle for contact radius calculation.


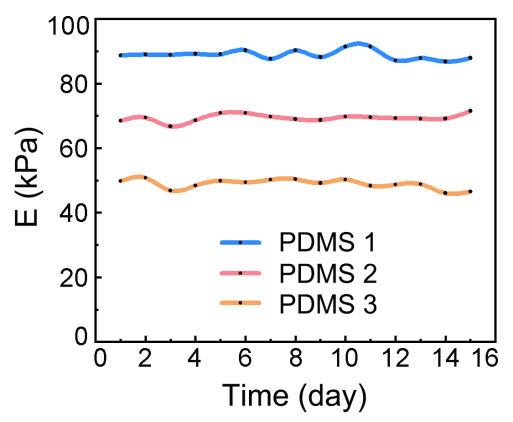


Figure S3. Variation in the measured modulus of PDMS over 15 consecutive days.
